# Combined strategy of maximal endoscopic endonasal resection and early radiation therapy for complex cystic and solid craniopharyngioma: operative video

**DOI:** 10.3171/2020.4.FocusVid.19963

**Published:** 2020-04-01

**Authors:** James K. Liu, Kevin Zhao, Jean Anderson Eloy

**Affiliations:** Departments of 1 Neurological Surgery and; 2 Otolaryngology–Head and Neck Surgery,; 3 Center for Skull Base and Pituitary Surgery, Neurological Institute of New Jersey, Rutgers University, New Jersey Medical School, Saint Barnabas Medical Center, RWJ Barnabas Health, Livingston and Newark, New Jersey

**Keywords:** extended endoscopic endonasal approach, craniopharyngioma, radiation therapy, combined approach, transplanum transtuberculum, surgical video

## Abstract

Craniopharyngioma is a rare and benign intracranial tumor of the sellar and suprasellar region. Historically, these tumors were mostly accessed through transcranial corridors and resected with microsurgical techniques. Endoscopic endonasal surgery has recently gained popularity in the treatment of these tumors and has shown at least comparable results to transcranial approaches. The endoscopic endonasal approach provides direct midline access through a transplanum transtuberculum corridor and gives excellent visualization of the undersurface of the optic chiasm to allow safe bimanual sharp dissection of the tumor from the hypothalamus. In this operative video, we demonstrate the case of a 56-year-old female who had a complex craniopharyngioma with solid and cystic components extending superolaterally into the right frontal lobe. This lesion was invasive and partially encased the right optic nerve, optic chiasm, and anterior communicating artery complex. Although a traditional transcranial approach could have been utilized, we elected for an endoscopic endonasal approach for a maximal safe near-total resection, preserving the neurovascular structures. The patient underwent radiation therapy with favorable regression of the residual tumor on subsequent imaging studies. This case illustrates the feasibility of a combined strategy of maximal safe endoscopic endonasal resection followed by early radiation therapy for a complex, invasive cystic and solid craniopharyngioma. The technical nuances of safe bimanual microsurgical dissection of tumor adhesions off of critical neurovascular structures are demonstrated.

The video can be found here: https://youtu.be/z0AINLpRZGs.

## Transcript

This is Dr. James Liu from Rutgers New Jersey Medical School. I will be demonstrating a combined strategy of maximal endoscopic endonasal resection followed by radiation therapy for a complex cystic and solid craniopharyngioma.

### 0:35 Patient history and physical examination

The patient is a 56-year-old female who presented to the emergency department with slight confusion, progressive headache and visual loss, increased appetite, and weight gain. She was awake and alert, oriented ×3, and following commands. However, she could not remember how she got to the hospital. Visual acuity exam revealed 20/150 on the right and 20/40 on the left with bitemporal hemianopsia. The remainder of her neurological exam was nonfocal. Endocrine workup showed normal anterior pituitary function and no diabetes insipidus.

### 1:11 Preoperative imaging

MRI showed a complex craniopharyngioma with a solid component in the suprasellar retrochiasmatic region associated with a large cyst extending into the right frontal lobe resulting in mass effect on the right lateral ventricle.

### 1:26 Surgical approach selection

We considered a variety of transcranial approaches, including the pterional and orbitozygomatic approach, also the endoscopic endonasal approach, and a combined transcranial/endonasal approach as well.

We eventually chose the endoscopic endonasal approach as the first stage because we felt that this would give us the best optic nerve decompression with better visualization of the hypothalamus and potential stalk preservation.

Our goal was to perform maximal safe resection, and possibly come back for a second-stage transcranial approach from above, if needed.

### 2:02 Patient positioning

After placing a lumbar drain, the patient is positioned supine in pins with the head rotated to the right approximately 15°. Image guidance is used along with visual and somatosensory evoked potentials. The nose and nares are prepped with betadine, and the right thigh is prepped for a fascia lata graft.

### 2:01 Phases of EEA: surgical team

The endoscopic endonasal approach can be divided into the following phases with the respective surgical teams.

### 2:29 Endoscopic endonasal approach: sinonasal phase

Using a 30° endoscope, the nasal mucosa is infiltrated with 1% lidocaine with 1:100,000 of epinephrine. The inferior and middle turbinates are lateralized, and the right middle turbinate is resected in this particular case to create a larger working corridor for the endoscope and the working instrument.

### 2:55 Endoscopic endonasal approach: nasoseptal flap harvest

A vascularized pedicled nasoseptal flap is harvested in the right nostril. The incision is made along the lateral nasal wall under the inferior turbinate and carried anteriorly along the floor of the nose toward the anterior septal incision. This maximizes the surface area of the nasoseptal flap. The flap is carefully dissected away from the nasal septum in a subperichondrial and subperiosteal fashion. Dissection is carried along the floor of the nose, separating the adhesive decussating fibers. Posteriorly, we can now visualize the right rostrum of the sphenoid sinus. The superior margin of the flap is incised anteroposteriorly, preserving the superior olfactory strip. The incision is carried toward the right sphenoid sinus, and the flap is mobilized inferiorly into the nasopharynx for storage until later use.

### 3:53 Endoscopic endonasal approach: sphenoidotomy and transplanum transtuberculum skull base exposure phase

The left sphenoid sinus is opened to complete the bilateral sphenoidotomy and a posterior septectomy is performed to allow adequate binostril access. The sphenoid sinus mucosa is removed to better visualize the bony landmarks of the skull base, and the sphenoidotomy is widened with a high-speed drill. It is important to drill down the septae of the sphenoid sinus with caution since these tend to guide you toward the cavernous carotid arteries.

The bony sellar floor is removed with a Kerrison rongeur, and the planum and tuberculum sellae is thinned down with a high-speed drill with copious irrigation. Once the tuberculum is reduced to eggshell thickness, the bone is outfractured with an upangled curette. It is important to visualize the medial optic canals bilaterally.

### 4:52 Dural opening

The dura is coagulated in the midline along with the intercavernous sinus. We then open the dura above and below the intercavernous sinus and incise the sinus into the diaphragma sella to expose the suprasellar cistern.

### 5:13 Resection of tumor phase

During the dural opening, the cyst cavity is entered and the yellow-greenish cystic fluid is encountered. Initial debulking of the tumor is performed with angled ring curettes to decompress the tumor. The tumor capsule is dissected away from the left internal carotid artery as it exits the distal dural ring. Care is taken to identify and preserve the superior hypophyseal perforators coming off the medial aspect of the carotid, as these tend to supply the optic apparatus.

The tumor capsule is carefully dissected away from the right optic nerve, and further debulking of the solid tumor contents is performed. Here, we identify a superior hypophyseal artery perforator coming off of the left ICA. It is important to resist the temptation to coagulate this perforator to avoid the risk of visual loss. Instead, sharp dissection is performed to dissect the perforator off the tumor capsule. The vessel courses posteriorly around the tumor and back to the optic apparatus.

We identify the pituitary stalk by gently lifting the tumor off of the normal gland. The portal striations of the stalk allow us to confirm this structure. The stalk is preserved by using sharp dissection to lyse tumor adhesions with bimanual microsurgical technique. The anterior inferior portion of the cyst wall is removed to provide better exposure into the suprachiasmatic region. It now becomes apparent that the tumor capsule partially encases the right optic nerve by coursing superiorly over the nerve and into the right frontal lobe cyst. Bimanual microsurgical sharp dissection is used to cut the cyst wall along the course of the right optic nerve and chiasm in order to separate the retrochiasmatic portion from the frontal lobe cyst.

The retrochiasmatic portion of the tumor is now dissected off the optic chiasm. The right tumor capsule is now dissected away from the right posterior communicating artery laterally, and off of the membrane of Liliequist inferiorly. Keeping the arachnoid of the Liliequist membrane intact helps protect the basilar artery and P1 perforators.

The endoscopic endonasal approach gives excellent visualization of the undersurface of the optic chiasm to allow safe bimanual sharp dissection of the tumor from the hypothalamus. The tumor is dissected away from the right aspect of the pituitary stalk where it meets the hypothalamus. We can now visualize the basilar artery apex posteriorly. We now roll the tumor away from the retrochiasmatic region and cut the remaining adhesions from the pituitary stalk. It is important to ensure that all adhesions have been released before delivering the tumor and to avoid the temptation of premature pulling of the tumor.

Final inspection shows the cavity of the frontal lobe cyst and microscopic adhesions to the anterior communicating artery complex. We determine that the cyst wall is beyond safe reach and dissection endonasally and that we have achieved the best maximal safe resection from below.

### 9:46 Multilayer reconstruction phase

Multilayered reconstruction is performed by initially placing a DuraGen inlay graft followed by an autologous fascia lata graft. The fascia lata is fashioned to cover the dural defect and a small margin of the bony edges. This is then supported by a layer of Surgicel. The vascularized pedicled nasoseptal flap is then mobilized and rotated into position over the dural closure. Care is taken to ensure that the flap is apposed to the bony skull base surface without any tension on the pedicle. The flap is then bolstered with Surgicel followed by gentamicin-soaked Gelfoam pledgets, and finally buttressed with a Merocel pack. The Merocel pack is left in for approximately 10–12 days after surgery and taken out in the office.

### 10:50 Postoperative course and postoperative imaging

Postoperatively, the patient was awake and alert, and following commands with an intact neurological exam. The mental status improved and anterior pituitary function was normal. There was transient diabetes insipidus which resolved after a few doses of DDAVP. Visual function improved to 20/20 on the right and 20/25 on the left with resolution of bitemporal hemianopsia. The lumbar drain was opened postoperatively at 5–10 cc per hour and there was no postoperative CSF leakage.

Postoperative day 1 MRI showed near-total resection of the tumor with collapse of the frontal lobe cyst wall and excellent decompression of the optic chiasm.

The MRI was repeated at 3 months, which showed further collapse of the right frontal lobe cyst which communicated directly into the suprasellar cistern. At this juncture, we decided to proceed with radiation therapy with IMRT instead of performing a second-stage transcranial approach for the cyst. At 16 months postoperatively, there was further regression of the remaining tumor and the patient remained recurrence free at 3.5 years postoperatively.

### 12:01 Conclusion

This case illustrates the combined strategy of maximal safe endoscopic endonasal resection followed by radiation therapy as an option for these complex craniopharyngiomas with neurovascular invasion.

## Supplemental Information

Supplemental Figs. 1 and 2

## References

[1] Fernandez-Miranda JC Gardner PA Snyderman CH Devaney KO Strojan P Suárez C Craniopharyngioma: a pathologic, clinical, and surgical review Head Neck 34 1036 1044 2012 21584897 10.1002/hed.21771

[2] Gardner PA Kassam AB Snyderman CH Carrau RL Mintz AH Grahovac S Outcomes following endoscopic, expanded endonasal resection of suprasellar craniopharyngiomas: a case series J Neurosurg 109 6 16 2008 18590427 10.3171/JNS/2008/109/7/0006

[3] Gardner PA Prevedello DM Kassam AB Snyderman CH Carrau RL Mintz AH The evolution of the endonasal approach for craniopharyngiomas J Neurosurg 108 1043 1047 2008 18447729 10.3171/JNS/2008/108/5/1043

[4] Koutourousiou M Gardner PA Fernandez-Miranda JC Tyler-Kabara EC Wang EW Snyderman CH Endoscopic endonasal surgery for craniopharyngiomas: surgical outcome in 64 patients J Neurosurg 119 1087 1357 2013 23909243 10.3171/2013.6.JNS122259

[5] Liu JK Christiano LD Patel SK Eloy JA Surgical nuances for removal of retrochiasmatic craniopharyngioma via the endoscopic endonasal extended transsphenoidal transplanum transtuberculum approach Neurosurg Focus 30 4 e14 2011 10.3171/2011.1.FOCUS10297 21456925

[6] Liu JK Sevak IA Carmel PW Eloy JA Microscopic versus endoscopic approaches for craniopharyngiomas: choosing the optimal surgical corridor for maximizing extent of resection and complication avoidance using a personalized, tailored approach Neurosurg Focus 41 6 e5 2016 10.3171/2016.9.FOCUS16284 27903113

