# Supplementary material for: Combined strategy of maximal endoscopic endonasal resection and early radiation therapy for complex cystic and solid craniopharyngioma: operative video
Source: Neurosurg Focus Video. 2020 Apr 1;2(2):V7. doi: 10.3171/2020.4.FocusVid.19963 (PMC9542377; doi:10.3171/2020.4.FocusVid.19963)
Supplement: Supplemental Figs. 1 and 2 [file 19963.Liu.NSVapr2020.supplmat.pdf]

Supplemental material

Combined strategy of maximal endoscopic endonasal resection and early radiation therapy for complex cystic and solid craniopharyngioma: operative video

James K. Liu, MD,[1–3] Kevin Zhao, DO,[1] and Jean Anderson Eloy, MD[1–3]

http://thejns.org/doi/abs/10.3171/2020.4.FocusVid.19963

DISCLAIMER *Neurosurgical Focus: Video* acknowledges that the following section is published verbatim as submitted by the authors and did not go through either the journal's peer-review or editing process.

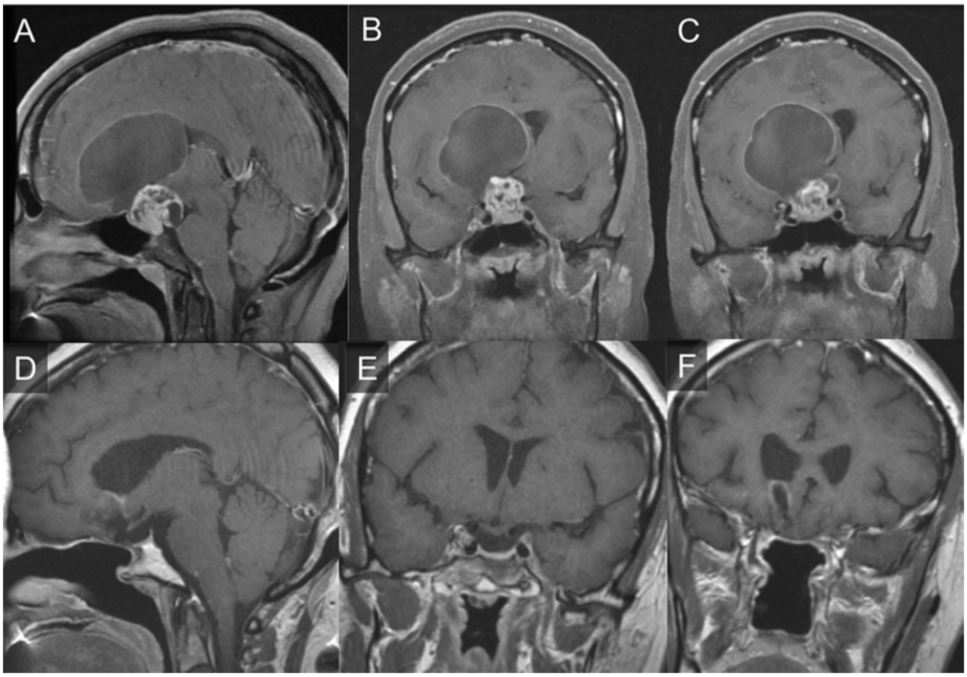

FIG. 1. Preoperative postgadolinium T1-weighted MRI sagittal (A) and coronal (B and C) views demonstrating a complex craniopharyngioma with a solid component in the retrochiasmatic space associated with a large cyst extending into the right frontal lobe. Postoperative postgadolinium T1-weighted MRI sagittal (D) and coronal (E and F) views after radiation therapy at 16 months showing regression of the right frontal lobe cyst with minimal residual tumor.

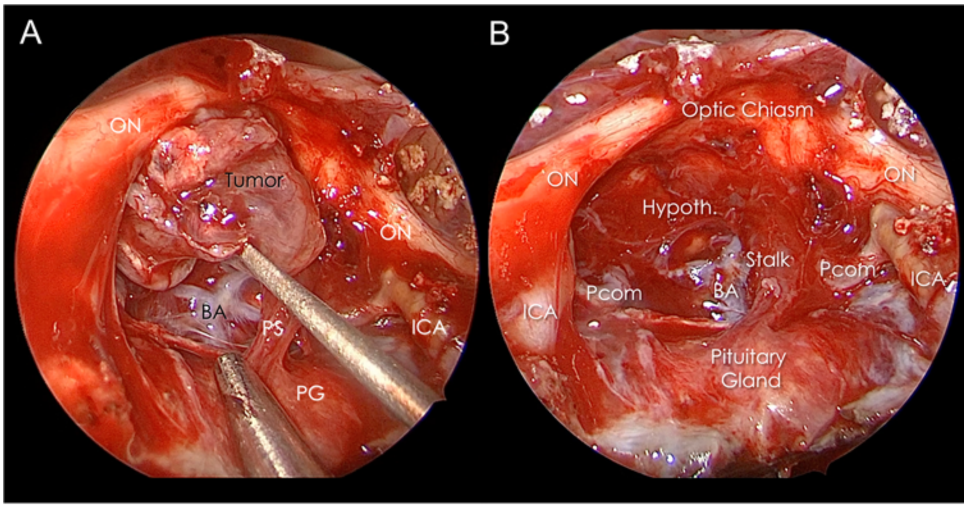

FIG. 2. A: Extended endoscopic view of solid craniopharyngioma in the retrochiasmatic space. The tumor is dissected off of the pituitary stalk (PS) and the basilar apex (BA) is visualized. B: Endoscopic view of the suprasellar retrochiasmatic region after near-total resection of the tumor. Hypoth. = hypothalamus; ICA = internal carotid artery; ON = optic nerve; Pcom = posterior communicating artery; PG = pituitary gland.
